# Inhibition of dipeptidyl peptidase-IV enzyme activity protects against myocardial ischemia-reperfusion injury in rats

**DOI:** 10.1186/s12967-014-0357-0

**Published:** 2014-12-13

**Authors:** Sarah Chua, Fan-Yen Lee, Tzu-Hsien Tsai, Jiunn-Jye Sheu, Steve Leu, Cheuk-Kwan Sun, Yung-Lung Chen, Hsueh-Wen Chang, Han-Tan Chai, Chu-Feng Liu, Hung-I Lu, Hon-Kan Yip

**Affiliations:** Department of Internal Medicine, Division of Cardiology, Kaohsiung Chang Gung Memorial Hospital, 123, Dapi Road, Niaosung District, Kaohsiung City, 83301 Taiwan R.O.C; Department of Surgery, Division of Thoracic and Cardiovascular Surgery, Kaohsiung Chang Gung Memorial Hospital, 123, Dapi Road, Niaosung District, Kaohsiung City, 83301 Taiwan R.O.C; Center for Translational Research in Biomedical Sciences, Kaohsiung Chang Gung Memorial Hospital, 123, Dapi Road, Niaosung District, Kaohsiung City, 83301 Taiwan R.O.C; Department of Emergency Medicine, E-DA Hospital, I-Shou University, 1, Yida Road, Jiaosu Village, Yanchao District, Kaohsiung City, 82445 Taiwan R.O.C; Department of Biological Sciences, National Sun Yat-Sen University, 70, Lienhai Road, Kaohsiung, 80424 Taiwan R.O.C; Institute of Shock Wave Medicine and Tissue Engineering, Kaohsiung Chang Gung Memorial Hospital and Chang Gung University College of Medicine, 123, Dapi Road, Niaosung District, Kaohsiung City, 83301 Taiwan R.O.C

**Keywords:** Ischemia-reperfusion injury, Dipeptidyl peptidase-IV (DPP4) enzyme, Sitagliptin, Inflammation, Oxidative stress, Heart function

## Abstract

**Background:**

We investigated whether attenuating dipeptidyl peptidase**-IV** (DPP4) enzyme activity protected rat heart from ischemia-reperfusion (IR) injury (40-min left anterior descending coronary artery ligation followed by 72 h reperfusion).

**Methods and results:**

Adult male Fischer 344 rats (n = 24) were equally divided into sham-control (WT-SC), WT-IR, and WT-IR-Sita (oral sitagliptin 400 mg/kg/day for 3 days) groups, whereas adult male DPP4-deficiency (DPP4^D^) rats (n = 16) were equally divided into DPP4^D^-SC and DPP4^D^-IR groups. Animals were sacrificed at 72 h after reperfusion with collection of heart specimens. Infarct area (H&E), collagen deposition (Sirius-red stain), fibrotic area (Masson's trichrome), and fluorescent-ROS intensity (H_2_DCFDA-labeling myocardium) of left ventricle were significantly higher in WT-IR than those in other groups, significantly higher in WT-IR-Sita and DPP4^D^-IR groups than in WT-SC and DPP4^D^-SC groups (all p < 0.001), but there was no difference between the latter two groups. Protein expressions of oxidative stress (oxidized protein), reactive oxygen species (NOX-1, NOX-2), inflammation (TNF-α, NF-κB, MMP-9, VCAM-1), apoptosis (mitochondrial Bax, cleaved caspase-3 and PARP), myocardial damage markers (cytosolic cytochrome-C, γ-H2AX), and number of inflammatory cells (CD14+, CD68+, CD40+ cells) showed a pattern identical to that of histological changes among all groups (all p < 0.005), whereas markers of anti-apoptosis (Bcl-2) and mitochondrial integrity (mitochondrial cytochrome-C) as well as left ventricular ejection fraction showed an opposite pattern (all p < 0.001). Protein expressions of anti-oxidants (HO-1, NQO-1), angiogenesis factors (SDF-1α, CXCR4), and glycogen-like-peptide-1-receptor were significantly higher inWT-IR-Sita and DPP4^D^-IR than those in other groups (all p < 0.001).

**Conclusion:**

Abrogation of DPP4 activity protects against myocardial IR injury and preserved heart function.

## Introduction

Myocardial ischemia-reperfusion (IR) injury contributes to adverse cardiac events after myocardial ischemia, cardiac surgery, cardiogenic shock, or circulatory arrest of different [1-5]. Despite reperfusion of the ischemic myocardium during primary percutaneous coronary intervention (PCI) is essential to minimize myocardial damage, reperfusion is also well-known for its deleterious effects [2] due to the generations of oxidative stress, reactive oxygen species (ROS), and vigorous inflammatory and immune reactions [1,6-10]. Of particular importance is that the ROS generated interacts with important proteins, such as ion channels, sarco-endoplasmic reticulum, calcium-release channels, and myofilament proteins which are associated with the excitation-contraction coupling. Alterations of the structures of these proteins can change their activities or their susceptibility to proteolysis. Studies have also shown that ROS are a major contributor to the opening of the mitochondrial permeability transition pore, causing the release of cytochrome *c* and other factors that can lead to hypercontracture and cell death [11]. This reaction ultimately causes loss of heart contractile function and alterations in the cardiovascular system [12,13]. Despite decades of intensive research, advanced pharmaco-therapeutic strategies, and state-of-the-art PCI procedure, there is still no effective treatment for myocardial IR injury.

Growing data have shown that glycogen-like peptide-1 (GLP-1) has inhibitory properties against inflammation and the generations of oxidative stress and ROS [14-17]. Additionally, GLP-1 receptor (GLP-1R) has been identified in many organs, including the brain, kidney, digestive organs, and probably also the [14,18,19]. This bioactive receptor has been shown to be upregulated in the setting of IR injury [14]. Dipeptidyl peptidase-IV (DPP4), a membrane-anchored ecto-protease, has been identified as leukocyte antigen CD26. Since GLP-1 is the substrate of DPP4 enzyme that can cleave circulating GLP-1, inhibition of DPP4 enzyme activity by sitagliptin, a drug for the treatment of type II diabetes mellitus and a DPP-IV inhibitor, has been reported to enhance the circulating GLP-1 level [14,20-23] which has also been found to be increased in DPP4-deficient animals and in acute kidney IR injury [14,22]. Sitagliptin, therefore, may offer cardiovascular protection through increasing circulating GLP-1 levels, thereby suppressing inflammation, oxidative stress, and the formation of atherosclerosis [20]. Accordingly, it is reasonable to believe that sitagliptin therapy may play a crucial role in protecting the heart from acute IR injury.

We have recently demonstrated extremely low DPP4 enzyme activity in DPP4-deficient animals [22]. In fact, DPP4-deficient rats are Fischer 344 rats with mutation of the DPP4 gene. Hence, it is rational to hypothesize that DPP4-deficient rats might be less vulnerable to IR-induced myocardial damage compared to the wide-type Fischer 344. We then further validated this hypothesis by investigating the effect of sitagliptin, a DPP4 inhibitor, on myocardial IRI in an experimental setting.

## Materials and methods

### Ethics

All animal experimental procedures were approved by the Institute of Animal Care and Use Committee at Chang Gung Memorial Hospital – Kaohsiung Medical Center (Affidavit of Approval of Animal Use Protocol No. 2010122405) and performed in accordance with the Guide for the Care and Use of Laboratory Animals (NIH publication No. 85–23, National Academy Press, Washington, DC, USA, revised 1996).

### Animal grouping and induction of acute myocardial ischemia-reperfusion injury

Pathogen-free, adult male Fischer 344 (i.e., wide type) rats (n = 24) weighing about 300-325 g (Charles River Technology, BioLASCO, Taiwan) were equally categorized into sham controls (WT-SC, n = 8), acute myocardial IR injury only (WT-IR, n = 8), and IR + oral sitagliptin (400 mg/kg) at post-IR 1, 24 and 48 h (WT-IR-Sita, n = 8). Additionally, adult male DPP4-deficiency rats (DPP4^D^, i.e., DPP4 mutant of Fischer 344; n = 16) weighing about 300-325 g (Charles River Technology, BioLASCO, Taiwan) were equally divided into sham control (DPP4^D^-SC, n = 8) and DPP4^D^-IR (n = 8). The dose-titration study which has been performed in our recent reports [14,22,24] identified that sitagliptin (400 mg/kg/per day) was the suitable dose for protecting animals in setting of critical limb ischemia and acute kidney ischemia-reperfusion injury. Accordingly, the dosage of sitagliptin (400 mg/kg) which was utilized in the present study was basic on our previous studies [14,22,24]. The time point of ischemia-reperfusion injury and sitagliptin treatment were basic on our previous reports with some modification [14,24].

All animals were placed in a supine position under anesthesia with 2.0% inhalational isoflurane on a warming pad at 37°C for the IR procedure. Under sterile conditions, the heart was exposed via a left thoracotomy. IR injury was induced in WT-IR, WT-IR-Sita, and DPP4^D^-IR animals by tightening left coronary artery for 40 minutes 3 mm distal to the margin of left atrium with a 7–0 prolene suture. Regional myocardial ischemia was verified by observing a rapid color change from pink to dull red over the anterior surface of the left ventricle and rapid development of akinesia and dilatation over the affected region. Rats receiving thoracotomy only without ischemia induction served as sham controls. The knot was then relieved after 40-minute ischemia, followed by 72 h reperfusion. The rats were sacrificed at 72 h after IR procedure, and hearts were harvested for individual study. The blood was collected from each animal for measuring the circulating level of GLP-1 by ELISA method using a commercialized kit. The dosages of sitagliptin to be utilized in this experiment were based on our recent report [14,16] with some modifications.

### Functional assessment by echocardiography

Transthoracic echocardiography was performed in each group prior to and on day 3 after myocardial IR induction. The procedure was performed by an animal cardiologist blind to the experimental design using an ultrasound machine (Vevo 2100, Visualsonics). M-mode standard two-dimensional (2D) left parasternal-long axis echocardiographic examination was conducted. Left ventricular internal dimensions [end-systolic diameter (ESD) and end-diastolic diameter (EDD)] were measured at mitral valve and papillary levels of left ventricle, according to the American Society of Echocardiography leading-edge method using at least three consecutive cardiac cycles. Left ventricular ejection fraction (LVEF) was calculated as follows: LVEF (%) = [(LVEDD^3^-LVEDS^3^)/LVEDD^3^] × 100%.

### Procedure and protocol for measurement of reactive oxygen species (ROS)

For determining the fluorescent intensity of ROS in myocardium, four additional animals were utilized in each study group. The protocol and detailed procedure of this test were based on our previous report [25]. By the end of study period (i.e., at 72 h after IR procedure), 2′,7′-dichlorodihydrofluorescin diacetate (H_2_DCFDA, Molecular Probes) was dissolved in DMSO at a concentration of 25 mg/mL. After being diluted with 50% ethanol to a final concentration of 2.5 mg/mL, it was administered intravenously at a dose of 6 μg/gm body weight to each animal. The rats were sacrificed 30 minutes following H_2_DCFDA administration.

The hearts were excised and sectioned at a level between the ligation and the apex. Totally three 2 mm-thick sections were obtained from each heart. All sections were examined under fluorescent microscope under a magnification of 100×. Both captured fluorescence and gray photos were assessed by using DP controller 2.1.1.183 (Olympus). Gray photos for measuring the fluorescence intensity were processed by using Image J 1.37v (National Institutes of Health, USA). Three gray photos from each section were randomly obtained, giving a total of nine photos for each animal. As compared with the area of increased fluorescence intensity (IFI), the baseline fluorescence intensity (BFI) [arbitrary unit/400 × high-power field (HPF)] was defined as that in myocardium without H_2_DCFDA. Six BFI areas were measured from each gray photo, from which three BFI areas were randomly chosen. The mean IFI and mean BFI were then calculated. The ratio of IFI to the BFI was determined as the relative fluorescence intensity.

### Western blot analysis of heart tissue

The procedure and protocol for Western blot analysis were based on our recent reports [14,22]. Briefly, equal amounts (50 μg) of protein extracts were loaded and separated by SDS-PAGE using acrylamide gradients. After electrophoresis, the separated proteins were transferred electrophoretically to a polyvinylidene difluoride (PVDF) membrane (Amersham Biosciences). Nonspecific sites were blocked by incubation of the membrane in blocking buffer [5% nonfat dry milk in T-TBS (TBS containing 0.05% Tween 20)] overnight. The membranes were incubated with the indicated primary antibodies [Bax (1: 1000, Abcam), cleaved poly (ADP-ribose) polymerase (PARP) (1:1000, Cell Signaling), caspase 3 (1: 1000, Cell Signaling), Bcl-2 (1:200, Abcam), (ICAM)-1 (1: 2000, Abcam), polyclonal antibodies against tumor necrotic factor (TNF)-α (1: 1000, Cell Signaling), nuclear factor (NF)-κB (1: 250, Abcam), MMP-9 (1:3000, Abcam), CXCR4 (1:1000, Abcam), stromal cell-derived factor (SDF)-1α (1:1000, Cell Signaling), NAD(P)H quinone oxidoreductase (NQO) 1 (1: 1000, Abcam), heme oxygense (HO)-1 (1: 250, Abcam), NADPH oxidase (NOX)-1 (1:1500, Sigma), NOX-2 (1:500, Sigma), cytosolic cytochrome C (1:2000, BD), mitochondrial cytochrome C (1:2000, BD), and GLP-1R (1:1000, abcam)] for 1 hour at room temperature. Horseradish peroxidase-conjugated anti-rabbit immunoglobulin IgG (1:2000, Cell Signaling) was used as a secondary antibody for one-hour incubation at room temperature. The washing procedure was repeated eight times within one hour. Immunoreactive bands were visualized by enhanced chemiluminescence (ECL; Amersham Biosciences) and exposed to Biomax L film (Kodak). For the purpose of quantification, ECL signals were digitized using Labwork software (UVP).

### Oxidative stress reaction in LV myocardium

The procedure and protocol for assessing the protein expression of oxidative stress have been described in details in our previous reports [26,27]. The Oxyblot Oxidized Protein Detection Kit was purchased from Chemicon (S7150). DNPH derivatization was carried out on 6 μg of protein for 15 minutes according to the manufacturer’s instructions. One-dimensional electrophoresis was carried out on 12% SDS/polyacrylamide gel after DNPH derivatization. Proteins were transferred to nitrocellulose membranes which were then incubated in the primary antibody solution (anti-DNP 1: 150) for 2 hours, followed by incubation in secondary antibody solution (1:300) for 1 hour at room temperature. The washing procedure was repeated eight times within 40 minutes. Immunoreactive bands were visualized by enhanced chemiluminescence (ECL; Amersham Biosciences) which was then exposed to Biomax L film (Kodak). For quantification, ECL signals were digitized using Labwork software (UVP). For oxyblot protein analysis, a standard control was loaded on each gel.

### Immunofluorescent (IF) and immunohistochemical (IHC) staining

IF staining was performed for the examinations of CD68+ and γ-H2AX cells in LV myocardium using respective primary antibodies based on our recent study [14,22,26]. Moreover, IHC staining was performed for examinations of CD14 and CD40 using respective primary antibodies as described [14,22,26]. Irrelevant antibodies were used as controls in the current study.

### Histological quantification of myocardial fibrosis/infarct and collagen deposition

The procedure and protocol was described in details in our previous report [26]. Briefly, hematoxylin and eosin (H&E) and Masson's trichrome staining were used for identifying the infarct area and fibrosis of LV myocardium, respectively. Three serial sections of LV myocardium in each animal at the same levels were prepared at 4 μm thickness by Cryostat (Leica CM3050S). The integrated area (μm^2^) of infarct area and fibrosis on each section were calculated using the Image Tool 3 (IT3) image analysis software (University of Texas, Health Science Center, San Antonio, UTHSCSA; Image Tool for Windows, Version 3.0, USA). Three randomly selected high-power fields (HPFs) (100 ×) were analyzed in each section. After determining the number of pixels in each infarct and fibrotic area per HPF, the numbers of pixels obtained from three HPFs were summated. The procedure was repeated in two other section for each animal. The mean pixel number per HPF for each animal was then determined by summating all pixel numbers and dividing by 9. The mean integrated area (μm^2^) of fibrosis in LV myocardium per HPF was obtained using a conversion factor of 19.24 (1 μm^2^ represented 19.24 pixels).

To analyze the extent of collagen synthesis and deposition, cardiac paraffin sections (6 μm) were stained with picrosirius red (1% Sirius red in saturated picric acid solution) for one hour at room temperature using standard methods. The sections were then washed twice with 0.5% acetic acid. The water was physically removed from the slides by vigorous shaking. After dehydration in 100% ethanol thrice, the sections were cleaned with xylene and mounted in a resinous medium. High power fields (×100) of each section were used to identify Sirius red-positive area on each section. Analytical sections of collagen deposition area in LV myocardium were identical to the description for the calculations of the infarct and fibrotic areas.

### Statistical analysis

Quantitative data are expressed as means ± SD. Statistical analysis was adequately performed by ANOVA followed by Bonferroni multiple-comparison post hoc test. SAS statistical software for Windows version 8.2 (SAS institute, Cary, NC) was utilized. A probability value <0.05 was considered statistically significant.

## Results

### The circulating level of GLP-1 at 72 h after IR injury

By day 3 after IR injury, the circulating level of GLP-1 was highest in DPP4^D^ + IR group and lowest in WT-SC, significantly higher in WT-IR-Sita group than that in WT-IR, but it exhibited no difference between WT-IR-Sita and DPP4^D^-SC groups, or between DPP4^D^-SC and WT-IR groups (Table 1). These findings suggest that DPP4^D^, IR injury, and sitagliptin treatment enhance the circulating level of GLP-1.

**Table 1:** **The circulating level of glycogen-like-peptide (GLP)-1 and echocardiography findings in five study groups of animals**

**Variables**	**Group 1 (n = 8)**	**Group 2 (n = 8)**	**Group 4 (n = 8)**	**Group 3 (n = 8)**	**Group 5 (n = 8)**	**P value** ^**†**^
Serum GLP-1 level (pg/ml)*	32.6 ± 2.3^a^	39.1 ± 3.1^b^	42.1 ± 4.7^b,c^	45.2 ± 2.9^c^	48.2 ± 4.1^d,c^	<0.0001
Baseline echocardiography						
LVEDd (cm)	6.8 ± 0.21	6.7 ± 0.21	6.4 ± 0.23	6.5 ± 0.24	6.5 ± 0.30	0.448
LVESd (cm)	3.4 ± 0.20	3.5 ± 0.18	3.4 ± 0.21	3.5 ± 0.20	3.4 ± 0.22	0.867
LEVF (%)	79.0 ± 1.8	79.0 ± 2.10	76.2 ± 3.70	77.0 ± 2.30	78.1 ± 2.80	0.662
Day 3 echocardiography*						
LVEDd (cm)	6.5 ± 0.24^a^	6.9 ± 0.37^b^	6.4 ± 0.38^a^	6.8 ± 0.34^b^	6.7 ± 0.28^b^	0.035
LVESd (cm)	3.3 ± 0.18^a^	5.7 ± 0.24^b^	3.4 ± 0.22^a^	4.3 ± 0.27^c^	4.2 ± 0.21^c^	<0.001
LVEF (%)	81.0 ± 1.60^a^	34.0 ± 4.2^b^	78.0 ± 3.40^a^	67.0 ± 3.70^c^	67 ± 3.30^c^	<0.001

Data are expressed as mean ± SD or %.

Group 1 = sham control [Wide type (WT)]; Group 2 = WT + ischemia-reperfusion (IR); Group 3 = DDP4-deficiency (DDP4^D^); Group 4 = DDP4^D^ + IR; Group 5 = WT + IR + sitagliptin.

LVEDd = left ventricular end-diastolic dimension; LVESd = left ventricular end systolic dimension; LVEF = left ventricular ejection fraction.

*indicates the blood sampling was performed at 72 h after the IR procedure.

^†^indicates by one-way ANOVA. Different letters (a, b, c) being used for group comparison, showing significant difference (<0.05) among different groups by Bonferroni’s multiple comparison procedure.

### Heart function prior to and at 72 h after IR injury

Table 1 showed the results of echocardiography findings. Prior to the IR procedure (i.e. day 0), the left ventricular ejection fraction (LVEF), left ventricular end-diastolic dimension (LVEDd) and left ventricular end-systolic dimension (LVESd) did not differ among the five groups. However, at 72 h after reperfusion, LVEF was significantly reduced, whereas LVEDd and LVESd were significantly increased in the WT-IR group as compared with the other groups. Additionally, LVEF was significantly reduced whereas LVEDd and LVESd were significantly increased in WT-IR-Sita and DPP4^D^-IR groups as compared to those of WT-SC and DPP4^D^-SC groups, but is showed no significant difference in these three parameters between the former two groups or between the latter two groups at 72 h after reperfusion. These findings suggest that DPP4^D^ and sitagliptin treatment preserved LV function and inhibited LV remodeling in the setting of IR-induced myocardial damage.

### ROS generation and histopathological findings at 72 h after IR procedure

**Figure 1:**
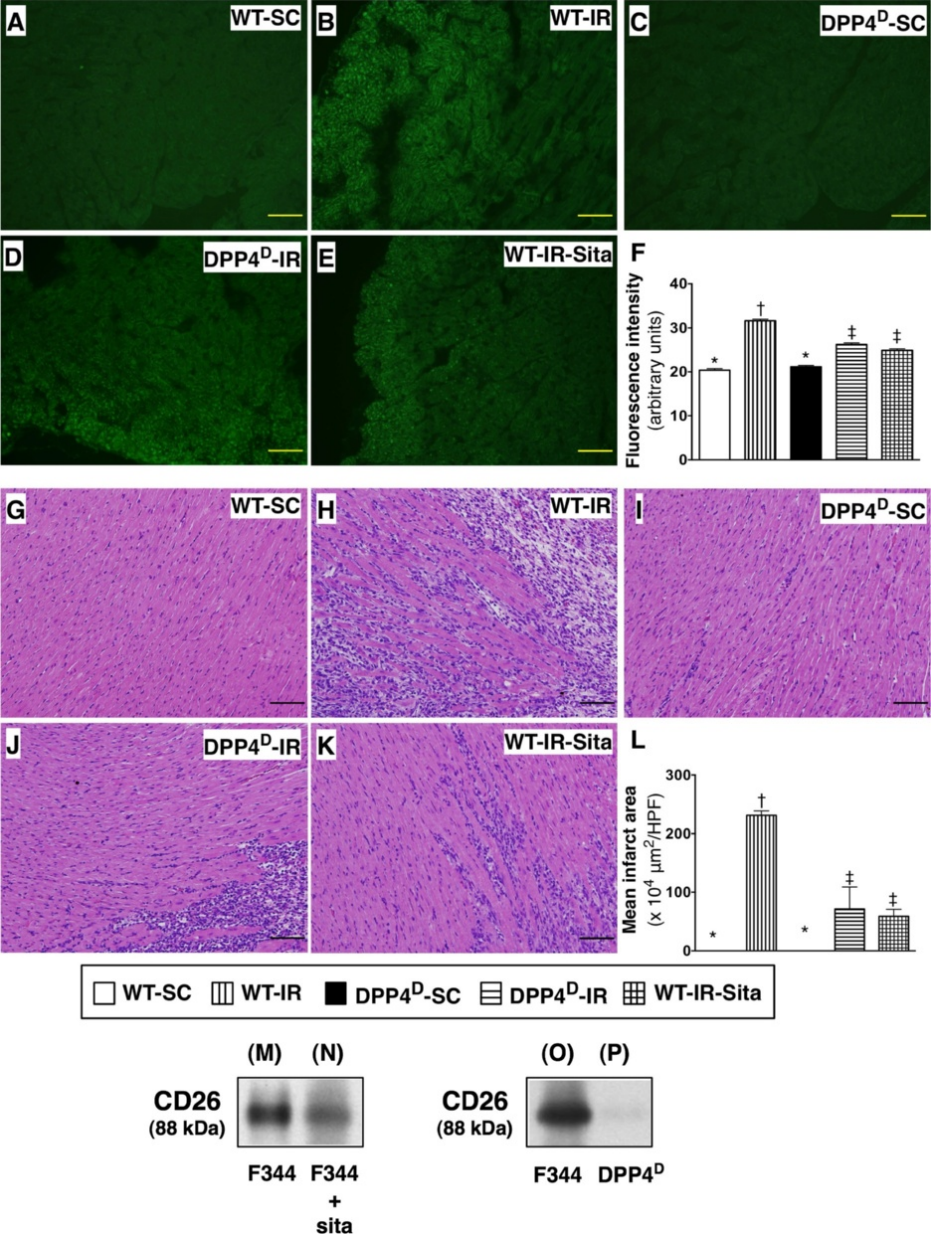
**Reactive oxygen species (ROS) generation in heart of live rats and pathological findings at 72 h after ischemia-reperfusion (IR) procedure. A** to **E)** Immunofluorescent microscopic findings (400×) of 2′,7′-dichlorodihydrofluorescin diacetate (H_2_DCFDA) stain for identifying the ROS generation in left ventricular (LV) myocardium. **F)** Analytic results of fluorescent intensity, p < 0.0001, * vs. other groups with different symbols (*, †, ‡). Scale bars in right lower corner represent 20 μm. **G** to **K)** Microscopic findings (100×) of H&E stain for identifying the infarct area in LV myocardium. **L)** Analytic results of infarct area, p < 0.0001, * vs. other groups with different symbols (*, †, ‡). Scale bars in right lower corner represent 100 μm. All statistical analyses were performed by one-way ANOVA, followed by Bonferroni multiple comparison post hoc test (n = 8). Symbols (*, †, ‡) indicate significance (at 0.05 level). WT-SC = wide type sham control; WT-IR = wide type + ischemia reperfusion (IR); DDP4^D^-SC = dipeptidyl peptidase-IV (DPP4) deficiency sham control; DDP4^D^-IR = DDP4^D^ + IR; WT-IR-Sita = wide type + IR + sitagliptin. HPF = high-power field. **M** and **N)** The Western blot results showed that the protein expression of circulating CD26, an indicator of DDP-4 activity, was remarkably suppressed in Fischer 344 rat with **(N)** than in without **(M)** sitagliptin treatment. **O** and **P)** The Western blot results showed that the protein expression of circulating CD26 in DDP-4 deficient rat **(P)** was lost as compared with that of circulating CD26 in Fischer 344 rat **(O)**.

To assess the role of DPP4^D^ and sitagliptin therapy in ROS expression in IR region of LV myocardium, the fluorescent intensity was quantified using H_2_DCFDA labeling of the myocardium as described in our previous study [25]. As expected, the relative fluorescence intensity was significantly increased in WT-IR than in other groups, significantly elevated in WT-IR-Sita and DPP4^D^-IR groups than in WT-SC and DPP4^D^-SC groups, but it showed no difference between the former two groups or between the latter two groups (Figure 1).

**Figure 2:**
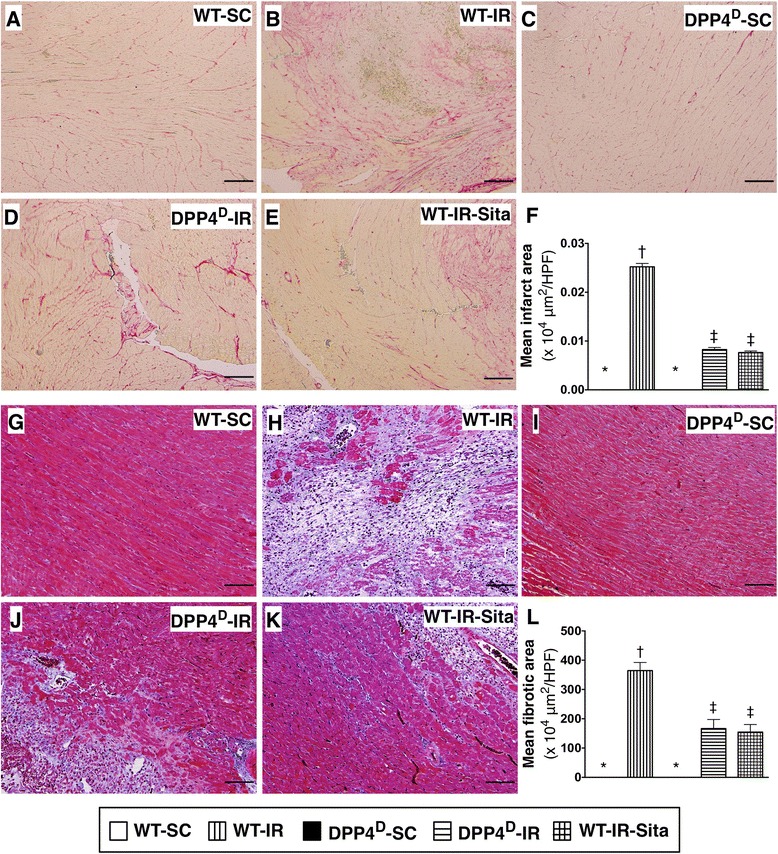
**Histopathological findings of left ventricular (LV) myocardium at 72 h after IR procedure. A** to **E)** Microscopic findings (100×) of Sirius red staining for identifying the collagen deposition in infarct area among five groups. **F)** Analytic results of collagen deposition, p < 0.0001, * vs. other groups with different symbols (*, †, ‡). Scale bars in right lower corner represent 100 μm. **G** to **K)** Microscopic findings (100×) of Masson's Trichrome staining for identifying the fibrotic area in LV myocardium. **L)** Analytic results of infarct area, p < 0.0001, * vs. other groups with different symbols (*, †, ‡). Scale bars in right lower corner represent 100 μm. All statistical analyses were performed by one-way ANOVA, followed by Bonferroni multiple comparison post hoc test (n = 8). Symbols (*, †, ‡) indicate significance (at 0.05 level). WT-SC = wide type sham control; WT-IR = wide type + ischemia reperfusion (IR); DDP4^D^-SC = dipeptidyl peptidase-IV (DPP4) deficiency sham control; DDP4^D^-IR = DDP4^D^ + IR; WT-IR-Sita = wide type + IR + sitagliptin. HPF = high-power field.

H&E staining demonstrated that the infarct area of left ventricle was largest in the WT-IR animals, significantly higher in WT-IR-Sita and DPP4^D^-IR groups than that in WT-SC and DPP4^D^-SC groups. However, the infarct area was not only similar between the former two groups, but it was also similar between the latter two groups (Figure 1). Additionally, Sirius-red staining demonstrated that the collagen deposition area in LV myocardium was identical to the result of H&E staining among the five groups of animals (Figure 2). Furthermore, Masson's trichrome staining showed that the fibrosis area in LV myocardium was identical to the finding of H&E staining among the five groups (Figure 2). These four histopathological findings imply that DPP4^D^ and sitagliptin treatment reduced ROS production and infarct size in the setting of IR-induced myocardial injury.

### Identifications of DNA damage marker and inflammatory cells in infarct region using IHC and IF stains at 72 h after reperfusion

**Figure 3:**
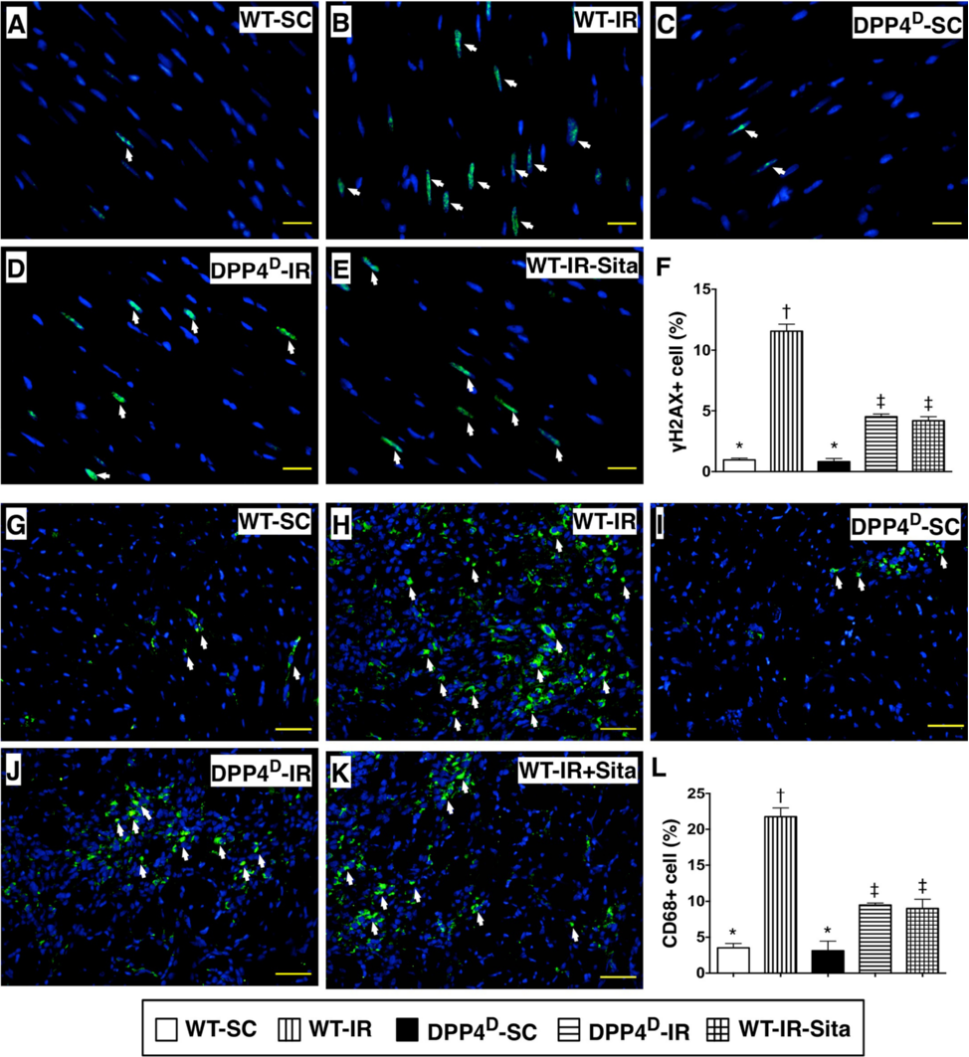
**Immunofluorescent (IF) stains of DNA damaged marker (γ-H2AX) and inflammatory cell (CD68+) infiltration in LV myocardium at 72 h after IR procedure. A** to **E)** IF microscopic findings (400×) of γ-H2AX+ cells (white arrows) in non-infarct area of LV myocardium. **F)** Comparison of number of γ-H2AX+ cells in non-infarct area among the five groups. p < 0.0001, * vs. other groups with different symbols (*, †, ‡). Scale bars in right lower corner represent 20 μm. **G** to **K)** IF Microscopic findings (200×) of CD68+ cells (white arrows) in infarct area. **L)** Comparison of number of CD68+ cells among the five groups. p < 0.0001, * vs. other groups with different symbols (*, †, ‡). Scale bars in right lower corner represent 50 μm. All statistical analyses were performed by one-way ANOVA, followed by Bonferroni multiple comparison post hoc test (n = 8). Symbols (*, †, ‡) indicate significance (at 0.05 level). WT-SC = wide type sham control; WT-IR = wide type + ischemia reperfusion (IR); DDP4^D^-SC = dipeptidyl peptidase-IV (DPP4) deficiency sham control; DDP4^D^-IR = DDP4^D^ + IR; WT-IR-Sita = wide type + IR + sitagliptin.

**Figure 4:**
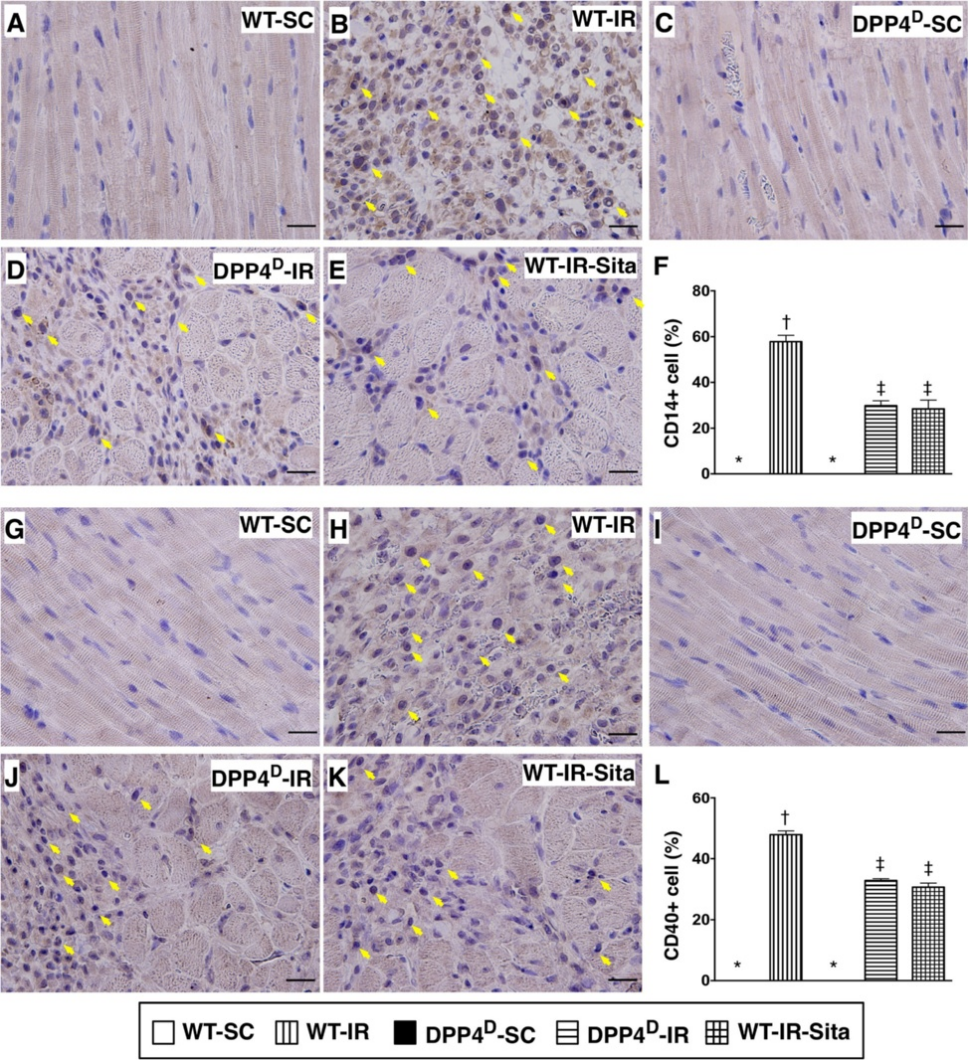
**Immunohistochemical (IHC) stains of inflammatory cell infiltrations in LV myocardium at 72 h after IR procedure. A** to **E)** Microscopic findings (400×) of IHC staining for identifying CD14+ cells (brown color) (yellow arrows) in infarct area. **F)** Comparison of number of CD14+ cells among the five groups. p < 0.0001, * vs. other groups with different symbols (*, †, ‡). Scale bars in right lower corner represent 20 μm. **G** to **K)** Microscopic findings (400×) of IHC stain for identifying CD40+ cells (brown color) (yellow arrows) in infarct area. **L)** Comparison of number of CD40+ cells among the five groups. p < 0.0001, * vs. other groups with different symbols (*, †, ‡). Scale bars in right lower corner represent 20 μm. All statistical analyses were performed by one-way ANOVA, followed by Bonferroni multiple comparison post hoc test (n = 8). Symbols (*, †, ‡) indicate significance (at 0.05 level). WT-SC = wide type sham control; WT-IR = wide type + ischemia reperfusion (IR); DDP4^D^-SC = dipeptidyl peptidase-IV (DPP4) deficiency sham control; DDP4^D^-IR = DDP4^D^ + IR; WT-IR-Sita = wide type + IR + sitagliptin.

The IF staining demonstrated that the expression of γ-H2AX-positive cells, an index of DNA damage, was significantly higher in WT-IR group than in other groups, and significantly higher in WT-IR-Sita and DPP4^D^-IR groups than in WT-SC and DPP4^D^-SC groups, but it showed no difference between the former two groups or between the later two groups (Figure 3). In addition, IF staining of LV infarct myocardium demonstrated that the pattern of changes in the number of CD68+ cells, another inflammation biomarker, was similar to that of γ-H2AX+ cells among the five groups (Figure 3). Furthermore, the IHC staining showed that the numbers of CD14+ and CD40+ cells in LV infarct myocardium, two indicators of inflammation, was identical to that of number of CD68+ cells among the five groups (Figure 4). These IHC/IF microscopic findings suggest that DPP4^D^ and sitagliptin treatment ameliorated DNA damage and inflammatory cell infiltration in the ischemic/infarct areas.

### Protein expressions of inflammatory biomarkers at 72 h after reperfusion

**Figure 5:**
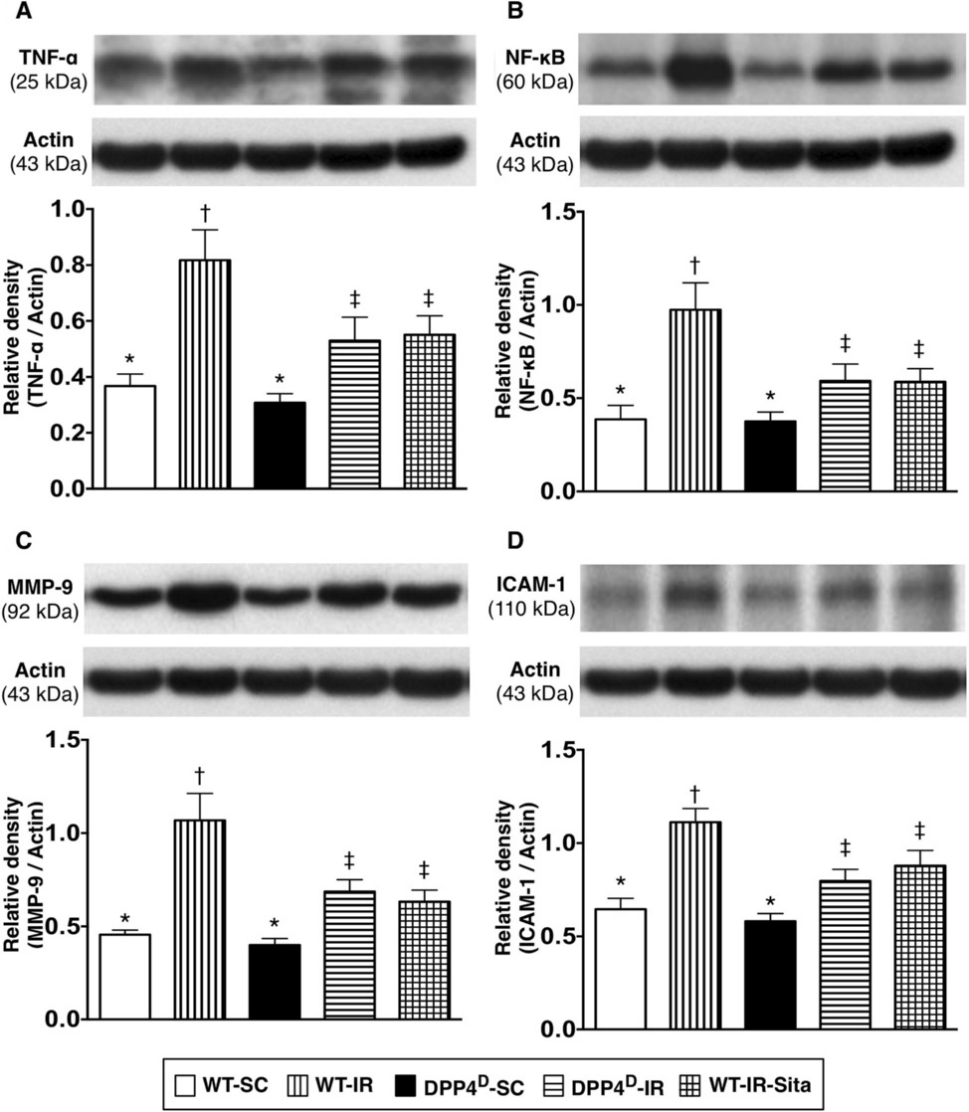
**Protein expressions of inflammatory biomarkers in LV myocardium at 72 h after IR procedure. A)** Protein expression of tumor necrosis factor (TNF)-α in LV myocardium. p < 0.001, * vs. other groups with different symbols (*, †, ‡). **B)** Protein expression of nuclear factor (NF)-κB in LV myocardium. p < 0.001, * vs. other groups with different symbols (*, †, ‡). **C)** Protein expression of matrix metalloproteinase (MMP)-9 in LV myocardium. p < 0.001, * vs. other groups with different symbols (*, †, ‡). **D)** Protein expression of intercellular adhesion molecule (ICAM)-1. p < 0.001, * vs. other groups with different symbols (*, †, ‡). All statistical analyses were performed by one-way ANOVA, followed by Bonferroni multiple comparison post hoc test (n = 8). Symbols (*, †, ‡) indicate significance (at 0.05 level). WT-SC = wide type sham control; WT-IR = wide type + ischemia reperfusion (IR); DDP4^D^-SC = dipeptidyl peptidase-IV (DPP4) deficiency sham control; DDP4^D^-IR = DDP4^D^ + IR; WT-IR-Sita = wide type + IR + sitagliptin.

The protein expressions of TNF-α (Figure 5-A), NF-κB (Figure 5-B), MMP-9 (Figure 5-C), and ICAM-1 (Figure 5-D), four indices of inflammation, were highest in the WT-IR group, significantly higher in the WT-IR-Sita and DPP4D-IR groups than those in the WT-SC and DPP4^D^-SC groups, but no significant difference was noted between the former two groups or between the latter two groups. These findings imply that DPP4^D^ and sitagliptin treatment attenuated inflammation at the molecular-cellular level.

### Myocardial ROS production, and protein expressions of ROS and oxidative stress at 72 h after reperfusion

**Figure 6:**
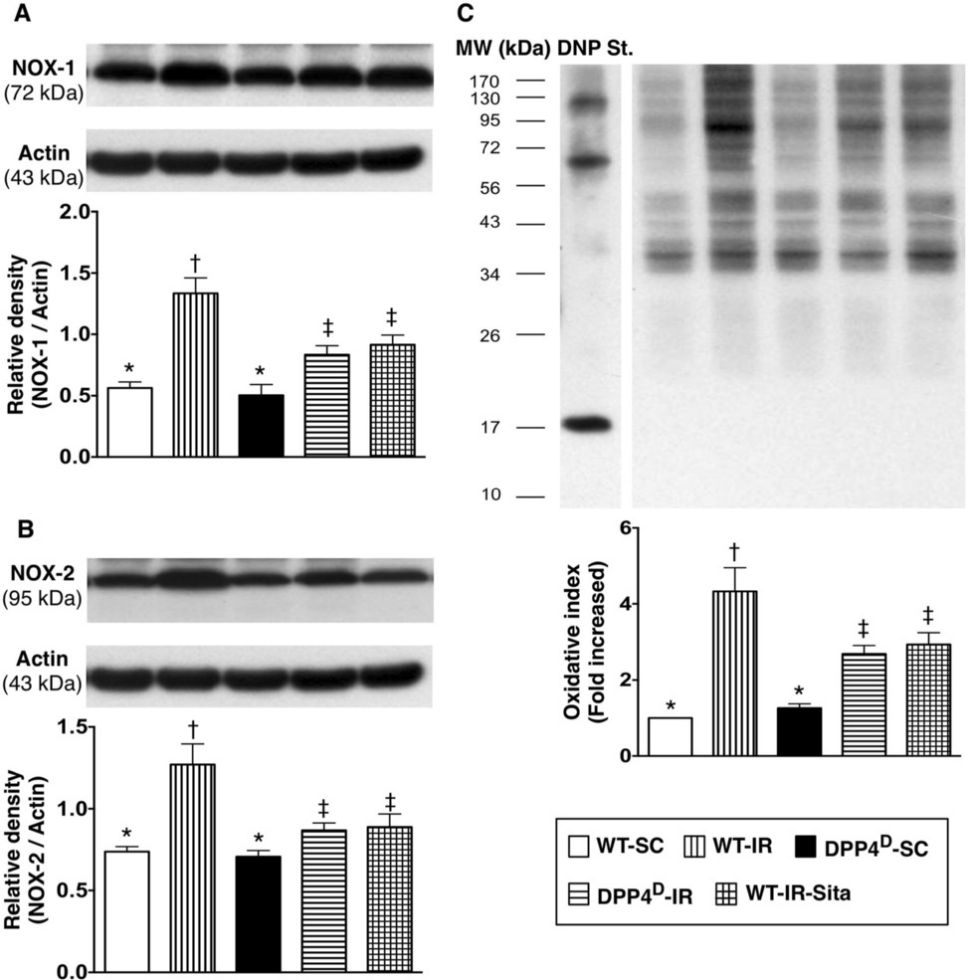
**Protein expressions of reactive oxygen species (ROS) and oxidative stress in LV myocardium at 72 h after IR procedure. A)** Protein expression of NOX-1 in LV myocardium. p < 0.001, * vs. other groups with different symbols (*, †, ‡). **B)** Protein expression of NOX-2 in LV myocardium. p < 0.001, * vs. other groups with different symbols (*, †, ‡). **C)** Protein expression of oxidative index (protein carbonyls) in LV myocardium. p < 0.0001, * vs. other groups with different symbols (*, †, ‡). (Note: right and left lanes shown on the upper panel represent control oxidized molecular protein and standard and protein molecular weight marker, respectively). DNP = 1–3 dinitrophenylhydrazone. All statistical analyses were performed by one-way ANOVA, followed by Bonferroni multiple comparison post hoc test (n = 8). Symbols (*, †, ‡) indicate significance (at 0.05 level). WT-SC = wide type sham control; WT-IR = wide type + ischemia reperfusion; DDP4^D^-SC = dipeptidyl peptidase-IV (DPP4) deficiency sham control; DDP4^D^-IR = DDP4^D^ + ischemia reperfusion; WT-IR-Sita = wide type + ischemia reperfusion + sitagliptin.

The protein expressions of NOX-1 (Figure 6-A) and NOX-2 (Figure 6-B), two ROS biomarkers, were highest in the WT-IR group, significantly higher in the WT-IR-Sita and DPP4D-IR groups than in the WT-SC and DPP4^D^-SC groups, but there was no significant difference between the former two groups or between the latter two groups. Consistently, the oxidized protein, an index of oxidative stress, showed an identical pattern compared with the protein expressions of ROS among the five groups (Figure 6-C). These findings indicate that DPP4^D^ and sitagliptin therapy suppressed the generation of ROS and alleviated the oxidative stress.

### Protein expressions of apoptosis, anti-apoptosis, and mitochondrial damage biomarkers at 72 h after reperfusion

**Figure 7:**
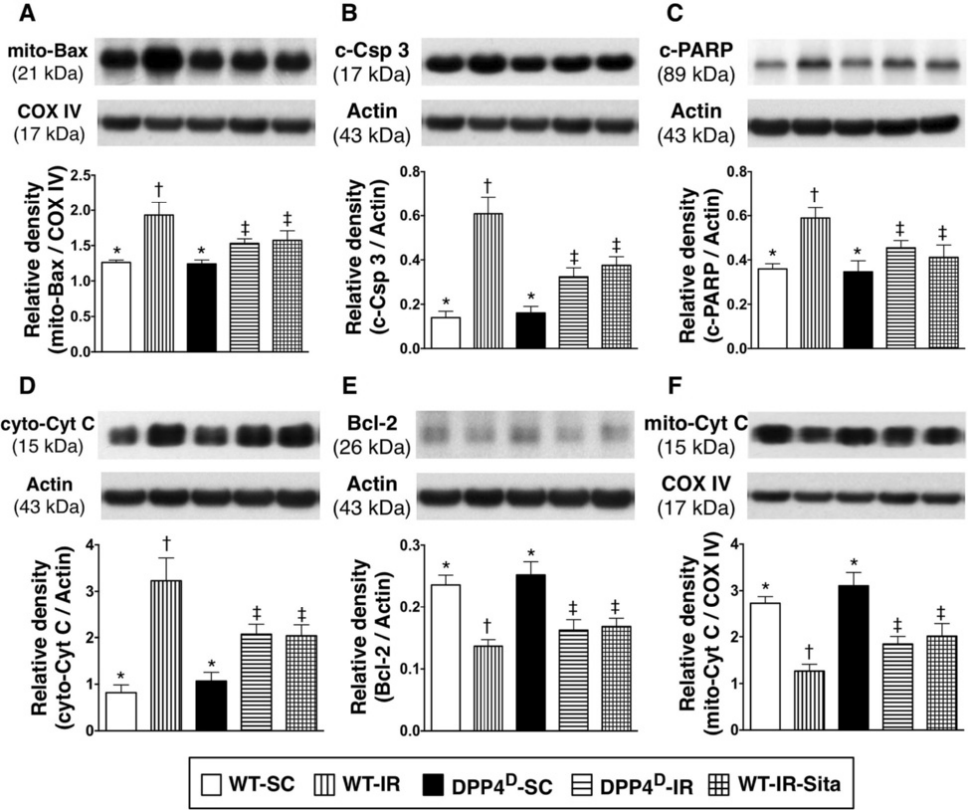
**Protein expressions of apoptotic, anti-apoptotic and mitochondrial damage biomarkers in LV myocardium at 72 h after IR procedure. A)** Protein expression of mitochondrial Bax in LV myocardium. p < 0.008, * vs. other groups with different symbols (*, †, ‡). **B)** Protein expression of cleaved caspase (Csp)-3 in LV myocardium. p < 0.001, * vs. other groups with different symbols (*, †, ‡). **C)** Protein expression of cleaved poly (ADP-ribose) polymerase (PARP) in LV myocardium. p < 0.01, * vs. other groups with different symbols (*, †, ‡). **D)** Protein expression of cytosolic cytochrome C (Cyto-Cyt C) in LV myocardium. p < 0.001, * vs. other groups with different symbols (*, †, ‡). **E)** Protein expression of Bcl-2 in LV myocardium. p < 0.01, * vs. other groups with different symbols (*, †, ‡). **F)** Protein expression of mitochondrial cytochrome C (Mito-Cyt C) in LV. p < 0.001, * vs. other groups with different symbols (*, †, ‡). All statistical analyses were performed by one-way ANOVA, followed by Bonferroni multiple comparison post hoc test (n = 8). Symbols (*, †, ‡) indicate significance (at 0.05 level). WT-SC = wide type sham control; WT-IR = wide type + ischemia reperfusion; DDP4^D^-SC = dipeptidyl peptidase-IV (DPP4) deficiency sham control; DDP4^D^-IR = DDP4^D^ + ischemia reperfusion; WT-IR-Sita = wide type + ischemia reperfusion + sitagliptin.

The protein expressions of mitochondrial Bax (Figure 7-A) and cleaved (i.e., active form) caspase 3 (Figure 7-B), and PARP (Figure 7-C), three indicators of apoptosis, were significantly higher in WT-IR group than in other groups, significantly higher in WT-IR-Sita and DPP4D-IR groups than in WT-SC and DPP4^D^-SC groups, but these parameters exhibited no difference between the former two groups or between the latter two groups. Additionally, the protein expression of cytosolic cytochrome C (Figure 7-D), an index of mitochondrial damage, showed an identical pattern compared to that of apoptotic biomarkers among the five groups. On the other hand, the protein expressions of Bcl-2 (Figure 7-E), an indicator of anti-apoptosis, and mitochondrial cytochrome C (Figure 7-F), an index of mitochondrial preservation, showed an opposite pattern compared to those of apoptotic biomarkers. These findings indicate that DPP4^D^ and sitagliptin treatment significantly reduced cellular apoptosis and mitochondrial damage.

### The protein expressions of GLP-1R and anti-oxidants at 72 h after IR procedure

**Figure 8:**
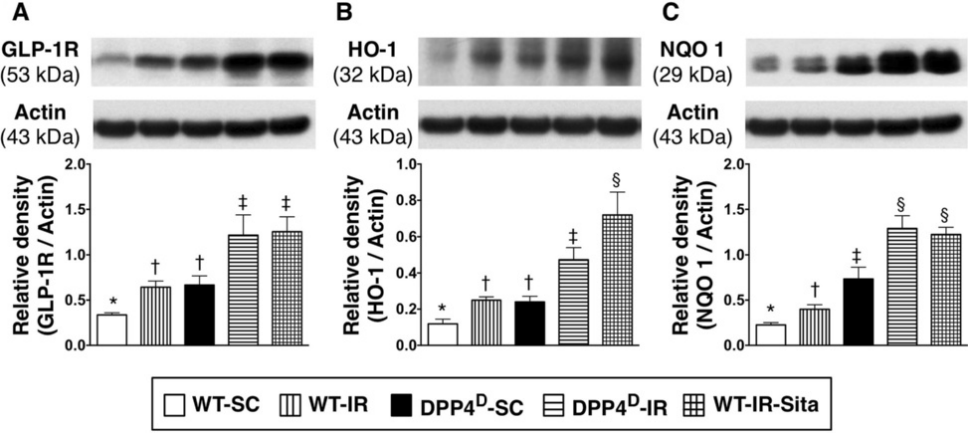
**Protein expressions of GLP-1 and antioxidants in LV myocardium at 72 h after IR procedure. A)** Protein expression of glycogen-like peptide-1 (GLP-1) in LV myocardium. p < 0.001, * vs. other groups with different symbols (*, †, ‡). **B)** Protein expression heme oxygense (HO)-1 in LV myocardium. p < 0.001, * vs. other groups with different symbols (*, †, ‡, §). **C)** Protein expression of NAD(P)H quinone oxidoreductase (NQO) 1 in LV myocardium. p < 0.001, * vs. other groups with different symbols (*, †, ‡, §). All statistical analyses were performed by one-way ANOVA, followed by Bonferroni multiple comparison post hoc test (n = 8). Symbols (*, †, ‡) indicate significance (at 0.05 level). WT-SC = wide type sham control; WT-IR = wide type + ischemia reperfusion; DDP4^D^-SC = dipeptidyl peptidase-IV (DPP4) deficiency sham control; DDP4^D^-IR = DDP4^D^ + ischemia reperfusion; WT-IR-Sita = wide type + ischemia reperfusion + sitagliptin.

The protein expression of GLP-1R was lowest in the WT-SC group, significantly lower in the WT-IR and DPP4^D^-SC groups than that in the WT-IR-Sita and DPP4^D^-IR groups, but there is no notable difference between the former two groups or the latter two groups (Figure 8-A). Moreover, the protein expression of HO-1, an indicator of anti-oxidant, was also lowest in the WT-SC group but highest in the WT-IR-Sita group, and significantly lower in the WT-IR and DPP4^D^ groups than that in the DPP4^D^-IR animals, but it showed no difference between the WT-IR and DPP4^D^ groups (Figure 8-B). Furthermore, the protein expression of NQO 1, another indicator of anti-oxidant, was lowest in the WT-SC group but highest in the WT-IR-Sita and DPP4^D^-IR groups, significantly lower in the WT-IR group than that in the DPP4^D^ group, but this parameter did not differ between the WT-IR-Sita and DPP4^D^-IR groups (Figure 8-C). These findings imply that DPP4^D^ and sitagliptin treatment offered anti-oxidative therapeutic effects.

### The expressions of angiogenesis factors and cardiac stem cells at 72 h after IR procedure

**Figure 9:**
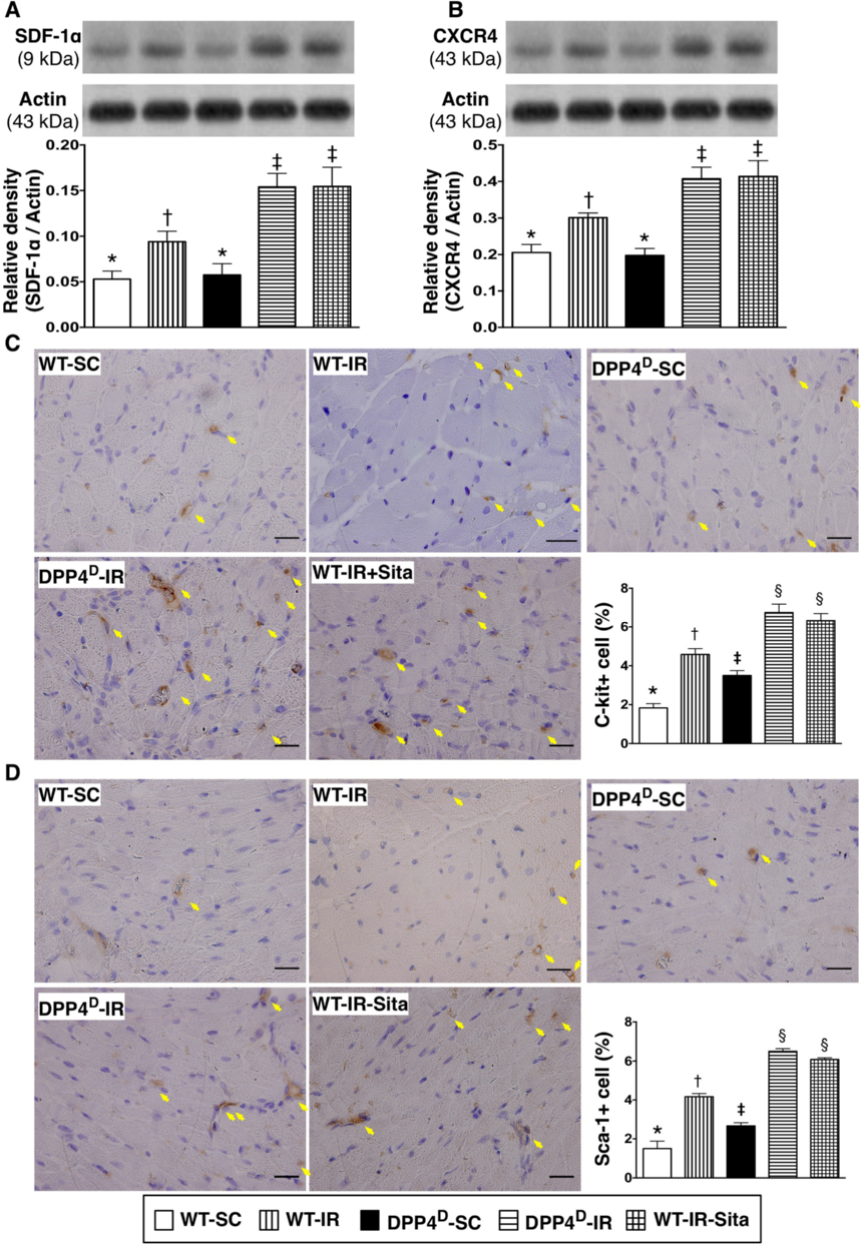
**Expressions of angiogenesis and cardiac progenitor cell biomarkers in LV myocardium at 72 h after IR procedure. A)** Protein expression of stromal cell-derived factor (SDF)-1α in LV myocardium. p < 0.01, * vs. other groups with different symbols (*, †, ‡). **B)** Protein expression of CXCR4 in LV myocardium. p < 0.01, * vs. other groups with different symbols (*, †, ‡). **C)** Expression of c-kit + cells in LV myocardium. <0.0001, * vs. other groups with different symbols (*, †, ‡, §). **D)** Expression of Sca-1+ cells in LV myocardium. <0.0001, * vs. other groups with different symbols (*, †, ‡, §). All statistical analyses were performed by one-way ANOVA, followed by Bonferroni multiple comparison post hoc test (n = 8). Symbols (*, †, ‡) indicate significance (at 0.05 level). WT-SC = wide type sham control; WT-IR = wide type + ischemia reperfusion; DDP4^D^-SC = dipeptidyl peptidase-IV (DPP4) deficiency sham control; DDP4^D^-IR = DDP4^D^ + ischemia reperfusion; WT-IR-Sita = wide type + ischemia reperfusion + sitagliptin.

The protein expressions of SDF-1α (Figure 9-A) and CXCR4 (Figure 9-B), two angiogenesis factors, were significantly lower in the WT-SC and DPP4^D^-SC groups than in other groups, significantly lower in WT-IR group than in that of DPP4^D^-IR and WT-IR-Sita groups, but is was no difference in the later two groups. Additionally, the numbers of c-kit + (Figure 9-C) and Sca-1+ (Figure 9-D) cells, two indicators of cardiac progenitor cells, were lowest in WT-SC group and highest in DPP4^D^-IR and WT-IR-Sita groups, and significantly higher in WT-IR group than in DPP4^D^-SC group. These findings may suggest that acute IR injury induced an elevation in intrinsic angiogenesis factors and proliferation of cardiac progenitor cells to protect myocardium against ischemia and that deletion of DPP4 enzyme activity might offer the most effective protection against IR-induced myocardial damage.

## Discussion

This study, which utilized DPP4-deficiency rats to clarify the therapeutic impact of sitagliptin, an oral hypoglycemic agent with distinctive property of inhibiting DPP4 enzyme activity, on the heart against IR injury in a rodent model, yielded several striking implications. First, not only were the size of LV infarct area and collagen deposition in infarct area significantly reduced, but LV function was also notably preserved in the WT-IR-Sita group than that in the WT-IR group. Second, the expressions of inflammatory, oxidative-stress, ROS, apoptotic, and myocardial damage biomarkers in infarct myocardium were remarkably reduced in the WT-IR-Sita animals than those in the WT-IR group. Consistently, the pattern of changes in these parameters in the DPP4^D^-IR group was identical to that in the WT-IR-Sita group. Third, the circulating GLP-1 levels and the expressions of anti-oxidants and GLP-1R in infarct myocardium were found to be markedly higher in the WT-IR-Sita group than that in the WT-IR group. In this regard, the changes of these biomarkers in DPP4^D^-IR animals showed a similar pattern compared to that in their WT-IR-Sita counterparts.

### Mechanisms underlying reduction of infarct size, suppression of LV remodeling, and preservation of LV function after sitagliptin treatment ― role of inhibiting DPP4 enzyme activity

Although several previous studies have shown that DPP4 inhibitors are capable of offering cardiovascular protection and preserving cardiac functions from myocardial ischemia [20,24,26-30], the precise mechanisms involved have not been extensively investigated. The most important findings in the present study is that, as compared with WT-SC, the infarct size, the fibrotic zone and collagen-deposited area of infarct myocardium and LV remodeling were substantially increased, whereas the cardiac function was notably reduced in the WT-IR animals and were remarkably restored in the WT-IR-Sita animals. To further elucidate whether the observation of cardiac protection offered by sitagliptin was through the inhibition of DPP4 enzyme activity, the extent of IR injury in DPP4-deficiency rats was investigated. As expected, the cardiac protective effects (i.e., reduction of infarct size, fibrotic area collagen-deposited region, and LV remodeling as well as preservation of cardiac function) in DPP4^D^-IR animals were found to be identical to that in the WT-IR-Sita group. Our results are consistent with those of previous studies [27,29] that demonstrated cardiac protection against ischemia-related myocardial injury through genetic deletion of DPP4.

**Figure 10:**
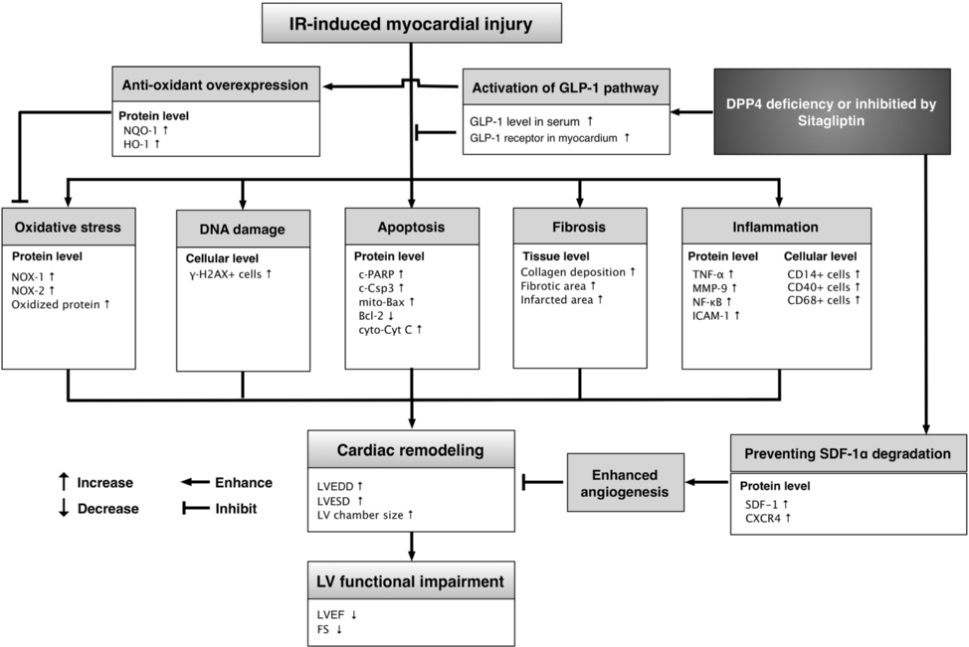
**Proposed mechanisms underlying the genetic deletion of DPP4 or therapeutic effect of sitagliptin on protecting heart from acute ischemia-reperfusion injury.** IR = ischemia-reperfusion; GLP-1 = glycogen-like peptide-1; DDP4 = dipeptidyl peptidase 4; HO-1 = heme oxygense-1; NQO 1 = NAD(P)H quinone oxidoreductase 1; c-PARP = cleaved poly (ADP-ribose) polymerase; c-Csp 3 = caspase 3; Cyto-Cyt C = cytosolic cytochrome C; TNF-α = tumor necrosis factor alpha; MMP-9 = matrix metalloproteinase 9; NF-κB = nuclear factor κB; ICAM-1 = intercellular adhesion molecule 1; SDF-1 = stromal cell-derived factor 1; LVEDd = left ventricular end diastolic dimension; LVESd = left ventricular systolic dimension; LVEF = left ventricular ejection fraction.

GLP-1 is the substrate of DPP4 that has been revealed to be capable of catalyzing the release of dipeptides from the N-terminus of GLP-1 which contains Pro or Ala in the third amino acid position [30]. Additionally, it is well recognized that sitagliptin can increase circulating GLP-1 levels via inhibiting DPP4 enzyme activity [14,20,21,23]. Accordingly, to elucidate whether the protective effect of sitagliptin against cardiac IR injury was through increasing the circulating level of GLP-1 and enhancing GLP-1R expression in LV myocardium, the levels of these two biomarkers were determined in the present study. As expected, the circulating level of GLP-1 and the protein expression of GLP-1R in myocardium were significantly increased in the WT-IR groups and further significantly increased in the WT-IR-Sita animals than in WT-SC animals. Intriguingly, in DPP4^D^ animals (i.e., DPP4 enzyme activity abrogated), the circulating level of GLP-1 was significantly higher as compared with that in WT-SC animals. Of particular importance is that the circulating GLP-1 level and protein expression of GLP-1R in LV myocardium were significantly increased in the DPP4^D^-IR group than those in the DPP4^D^-SC group. However, it showed no difference between the WT-IR-Sita and DPP4^D^-IR animals. These findings not only suggest that IR injury could induce intrinsic protective mechanism, but also support that augmentation of circulating GLP-1 level and GLP-1R expression in ischemic organ through sitagliptin treatment or DPP4 genetic deletion is one of the mechanisms involved in preserving heart function in the setting of IR injury. Our recent study [14] has shown that pharmacological inhibition of DPP4 by sitagliptin protected the kidney from IR injury mainly through increasing the endogenous level of GLP-1. In this way, our present finding is consistent with that of our recent report [14]. The proposed mechanisms by which through genetic deletion of DPP4 or sitagliptin therapy preserved heart function in a rodent model have been summarized in Figure 10.

### Up-regulating down-stream signalings of inflammatory reaction and the generations of oxidative stress and ROS in ischemia-reperfusion injury ― role of GLP-1 receptor pathway

Our previous experimental [14,24,26,31] have clearly shown that inflammation, ROS (i.e., oxygen free radicals), and oxidative stress, which are deleterious contributors to mitochondrial damage and cellular apoptosis, fibrosis and death as well as the propagation of organ dysfunction, were markedly up-regulated in the setting of ischemic organ dysfunction. The findings are consistent with those of the present study that demonstrated significant up-regulations in inflammation, ROS and oxidative stress, as well as apoptosis in the WT-IR animals compared with those in the WT-SC and DPP4^D^-SC animals. One distinctive finding is that these parameters were substantially down-regulated and the anti-apoptotic markers were notably up-regulated in the WT-IR-Sita animals. This is also in concert with the finding of our recent study that demonstrated remarkable inhibition of the expressions of inflammation, ROS, and oxidative stress in the settings of acute kidney injury and critical limb ischemia after sitagliptin treatment [14,24]. Another intriguing finding is that there was no difference in these parameters between the WT-IR-Sita and DPP4^D^-IR animals. Therefore, the findings suggest that sitagliptin suppressed inflammatory reaction, oxidative stress (i.e., the generations of ROS), and cellular apoptosis through the up-regulation of circulating GLP-1 and the interaction between GLP-1 and GLP-1R (i.e., GLP-1R pathway) on cardiomyocytes in the setting of cardiac IR injury. Accordingly, our findings also strengthen those of previous studies that demonstrated cardiovascular protective effect of DPP4 inhibitor/DPP4-deficiency through attenuating the productions of ROS [16] and oxidative stress [32].

### Inhibiting damaging effect of ROS and oxidative stress on myocardium through GLP-1-GLP-1R interacting pathway ― Up-regulation of anti-oxidant production

Anti-oxidants have been fully accepted as scavengers of ROS that alleviate oxidative stress at molecular-cellular level, especially in ischemia-related organ damage. Our recent study has shown that both sitagliptin and extendin-4 (i.e., an analogs of GLP-1) treatment offered effective cytoprotection through up-regulating the production of anti-oxidants and reducing the tissue levels of ROS in acute kidney IR injury [14]. A principal finding in the present study is that the protein expressions of antioxidants (HO-1, NQO 1) was markedly higher in WT-IR and DDF4^D^-SC animals than in WT-SC animals and the levels of these biomarkers were further markedly elevated in the WT-IR-Sita and the DPP4^D^-IR animals. The findings of high-level anti-oxidant production in DPP4^D^-IR animals imply that sitagliptin offers cardioprotective effect via enhancing the generation of anti-oxidants that alleviate the deleterious effect of ROS/oxidative stress on cardiomyocytes.

### Enhanced angiogenesis in infarct myocardium ― role of sitagliptin and deletion of DPP4 enzyme activity

A previous study has demonstrated that inhibition of DPP4 activity by enalapril enhanced the mobilization of endothelial progenitor cell (EPC) from bone marrow to circulation in the setting of critical limb ischemia [33]. Similarly, our recent studies have revealed that sitagliptin therapy augmented EPC mobilization from bone marrow to circulation and enhanced angiogenesis in ischemic regions [14,24]. An important finding in the current study is that, as compared with WT-SC and DPP4^D^-SC animals, the protein expression of angiogenesis factors (i.e., CXCR4 and SDF-1α) and the number of cardiac stem cells (i.e., c-kit and Sca-1) in infarct area were significantly higher in WT-IR animals. These findings may suggest that the ischemic stimulus triggered an intrinsic response in tissue/organ for self-protection. Intriguingly, these biomarkers were found to be notably higher in WT-IR-Sita than in WT-IR group. These findings corroborate those of our recent studies [14,24]. Of importance is that the expressions of these biomarkers in infarct LV did not differ between the WT-IR-Sita and DPP4^D^-IR animals. Our findings, in addition to supporting those of previous studies [33], also highlight that enhancement of angiogenesis and cardiac stem cell in ischemic area is mainly through the abrogating DPP4 activity. Therefore, our findings suggest that sitagliptin therapy and deletion of DPP4 may offer benefit in preserving cardiac function through restoring blood flow and regeneration of lost myocardium by cardiac stem cells.

### Study limitations

This study has limitations. First, this study did not investigate the impact of inhibition of DPP4 activity on long-term outcome of the animals after IR procedure. Second, although the dosage of sitagliptin to be utilized was based on our previous report [14], this study did not investigate which was the optimal sitagliptin dosage for the best myocardial protecting in the present IR setting based on pilot experiments.

In conclusion, inhibition of DPP4 activity protected the heart against IR injury and left ventricular remodeling and preserved heart function through down-regulating inflammation, oxidative stress, ROS generation, and cell apoptosis as well as up-regulating antioxidant production and angiogenesis.
